# Genome Sequence of *Devriesea agamarum*, Isolated from Agamid Lizards with Dermatitis

**DOI:** 10.1128/genomeA.00949-15

**Published:** 2015-08-20

**Authors:** Roel Haesendonck, Filip Van Nieuwerburgh, Freddy Haesebrouck, Dieter Deforce, Frank Pasmans, An Martel

**Affiliations:** a Department of Pathology, Bacteriology and Avian Diseases, Faculty of Veterinary Medicine, Ghent University, Merelbeke, Belgium; b Faculty of Pharmaceutical Sciences, Laboratory for Pharmaceutical Biotechnology, Ghent University, Ghent, Belgium

## Abstract

We report the genome sequence of *Devriesea agamarum* strain IMP2, isolated from the liver of a female *Agama impalearis*. This actinobacterium is associated with septicemia and dermatitis in agamid lizards. Availability of this genome sequence will contribute to the understanding of this pathogen’s virulence.

## GENOME ANNOUNCEMENT

Dermal diseases are frequently occurring problems in captive reptiles and are often associated with bacteria, especially coryneforms (1). *Devriesea agamarum*, an actinobacterium, was shown to be the causative agent in agamid lizards suffering from dermatitis and/or septicemia (2–5). Besides, the bacterium has also been isolated from the oral cavity of healthy bearded dragons (*Pogona vitticeps*), which can act as a pathogen source for more susceptible lizard species (6).

Here, we report the genome sequence of *D. agamarum* strain IMP2, isolated from the liver of a female *Agama impalearis*, which also showed dermal lesions.

Roche GS-FLX titanium libraries were generated, using 5 µg of the purified DNA sample. The DNA was fragmented by nebulization, followed by a double solid-phase reversible immobilization bead capture size selection with Ampure beads (Agencourt Bioscience) to generate DNA fragments of 400 to 1,500 bp in length. Selected fragments were end repaired and ligated to 454 sequencing adapters. A single-stranded library was then generated according to the Roche GS FLX Titanium general library preparation method manual (version October 2008). This library was used to perform an emulsion PCR according to the Roche GS FLX titanium emPCR method manual (version October 2008). The resulting bead library was sequenced on a Roche GS-FLX system following the GS FLX Titanium sequencing method manual (version October 2008), generating 411,000 reads.

The GS *de novo* Assembler version 2.6 was used to perform a *de novo* genome assembly using the GS FLX reads. More than 99% of the reads were assembled into 6 relevant contigs, with an *N*_50_ contig size of 2,321,398 bp. The total consensus sequence was 2,953,346 bp, with an average sequencing coverage of 55×. Illumina mate-paired libraries were generated using 10 µg genomic DNA following the Illumina mate-paired library v2 sample preparation guide (version November 2009). The resulting library was sequenced on an Illumina GAIIx flow cell following the Illumina genome analyzer user guide (version Rev. A, August 2009), generating 18,290,000 2 × 36-bp reads with an insert size of 3,000 bp.

The Illumina mate-paired reads were used to scaffold the 6 GS *de novo* Assembler contigs using SSPACE Basic version 2.0 (7), yielding 3 scaffolds with an *N*_50_ scaffold size of 2,763,216. The 3 remaining scaffolds were assembled into one single genome using a primer-based genomic walking method (8). A DNA Walking SpeedUp premix kit (Seegene, Eschborn, Germany) was used according to the manufacturer’s instructions and generated PCR amplicons spanning the gaps between the scaffolds. The gaps between the 3 scaffolds were all less than 59 bp and were sequenced across with a single forward and reverse Sanger sequencing reaction.

The availability of the genome sequence of *D. agamarum* will provide useful information to identify genes involved in this pathogen’s virulence and its evolution and adaptation to agamid lizard species.

### Nucleotide sequence accession numbers.

The genome sequence of the *Devriesea agamarum* type strain IMP2 has been deposited in the European Nucleotide Archive (ENA) under the accession number LN849456 (project identification number PRJEB9356).
